# Avascular Necrosis as a Sequela of COVID-19: A Case Series

**DOI:** 10.7759/cureus.35368

**Published:** 2023-02-23

**Authors:** Sarthak Parikh, Osmanny Gomez, Ty Davis, Zachary Lyon, Arturo Corces

**Affiliations:** 1 Dr. Kiran C. Patel College of Osteopathic Medicine, Nova Southeastern University, Fort Lauderdale, USA; 2 Department of Orthopedic Surgery, Larkin Community Hospital, Miami, USA

**Keywords:** coronavirus, covid-19, glucocorticoid therapy, glucocorticoid, avascular necrosis (avn)

## Abstract

Avascular necrosis (AVN) is a degenerative bone condition characterized by cellular death and bone collapse from compromised subchondral blood circulation. AVN begins with vascular interruption, hypertension, intravascular occlusion, or extravascular compression which reduces bone circulation. Although corticosteroids are frequently used to treat acute COVID-19 infections, patients are prone to its side effects, particularly AVN. Furthermore, COVID-19 produces coagulopathies, specifically hypercoagulability, that may contribute to venous thrombosis, which may serve as the impetus of AVN. While the literature discussing COVID-19, AVN, and corticosteroid use is not conclusive, patients being treated with corticosteroids for COVID-19 are at an increased risk for AVN possibly due to the combination of COVID-19 infection and corticosteroid use, or the use of high-dose steroids alone. The purpose of this case series is to elucidate AVN as a long-term sequalae of COVID-19, describe our management of COVID-19 and steroid-induced AVN, and discuss the current literature regarding AVN and COVID-19. Three patients hospitalized for COVID-19 infections were treated with corticosteroids and subsequently developed AVN. All patients, but one, had multiple sites of infarction and were treated with core decompression in the hip where there was no collapse of the subchondral bone. One of these patients had multiple infarcts in bilateral femoral heads, femoral shafts, and knees. This patient had a history of end-stage renal disease, and, therefore, total knee replacement was postponed until medical clearance. Core decompression was performed on the femoral head that showed no collapse to delay osteoarthritis of the hip. Multiple articles in the current literature support the idea that the combination of COVID-19 and corticosteroid use increases the risk of AVN and reduces the onset of COVID-19-related respiratory symptoms. The patient cases discussed in this case series suggest a possible association between COVID-19, corticosteroid use, and AVN.

## Introduction

Avascular necrosis (AVN) is a degenerative bone condition characterized by cellular death and bone collapse from compromised subchondral blood circulation. AVN begins with vascular interruption, hypertension, intravascular occlusion, or extravascular compression which reduces bone circulation. This necrotic process leads to disproportional rates of old bone resorption and new bone production that causes trabecular loss, subchondral fracture, and joint incongruity. It is this remodeling process that causes the clinical manifestations of AVN, rather than the necrosis itself [1].

The clinical manifestations depend on the location of AVN, which usually occurs in the anterolateral femoral head but can also affect many areas in the body such as the femoral condyle, humeral head, proximal tibia, vertebrae, and the small bones of the hands and feet [2]. Early in the disease course patients can be asymptomatic [2]. As AVN progresses, patients may have pain exacerbated by activity that eventually becomes nocturnal and persists at rest. Initial evaluation with X-ray may provide some evidence of AVN; however, MRI with contrast remains the gold standard for diagnosis. As the disease progresses, diagnostic studies will demonstrate the collapse of the subchondral surface with severe degenerative changes [1]. Some medications and drugs such as glucocorticoids, bisphosphonate, alcohol, or smoking can increase a patient’s risk of AVN. Conditions such as systemic lupus erythematosus, sickle cell, Gaucher’s disease, decompression disease, radiation therapy, or trauma can also predispose patients to develop AVN [2]. Recent literature suggests an association between COVID-19 and AVN in various joints in the body including the hip, vertebrae, knee, and jaw [3-6].

Corticosteroids are a widely used treatment for acute COVID-19 infections and work by reducing inflammation and suppressing the immune response leading to the cytokine storm that manifests as acute respiratory distress syndrome, disseminated intravascular coagulation, and multiple organ failure [7]. However, patients are also susceptible to the complications of corticosteroids, specifically AVN [7]. Although COVID-19 mainly affects the pulmonary and cardiovascular system, its destruction of alveolar epithelium can lead to the development of viremia affecting virtually any part of the body [8]. This predisposes patients to the long-term sequelae of COVID-19, which includes musculoskeletal involvement such as muscle weakness, fatigue, and pain [9]. Moreover, COVID-19 is known to produce coagulopathies, specifically hypercoagulability, that may contribute to venous thrombosis, which serves as the impetus of AVN [10].

While the literature discussing COVID-19, AVN, and corticosteroid use is inconclusive, patients being treated with corticosteroids for COVID-19 are at an increased risk for AVN possibly due to the combination of COVID-19 infection and corticosteroid use, or the use of high-dose steroids alone. The purpose of this case series is to elucidate AVN as a possible long-term sequela of COVID-19, describe our management of COVID-19 and corticosteroid-induced AVN, and discuss the current literature regarding AVN and COVID-19.

## Case presentation

Case one

**Figure 1:**
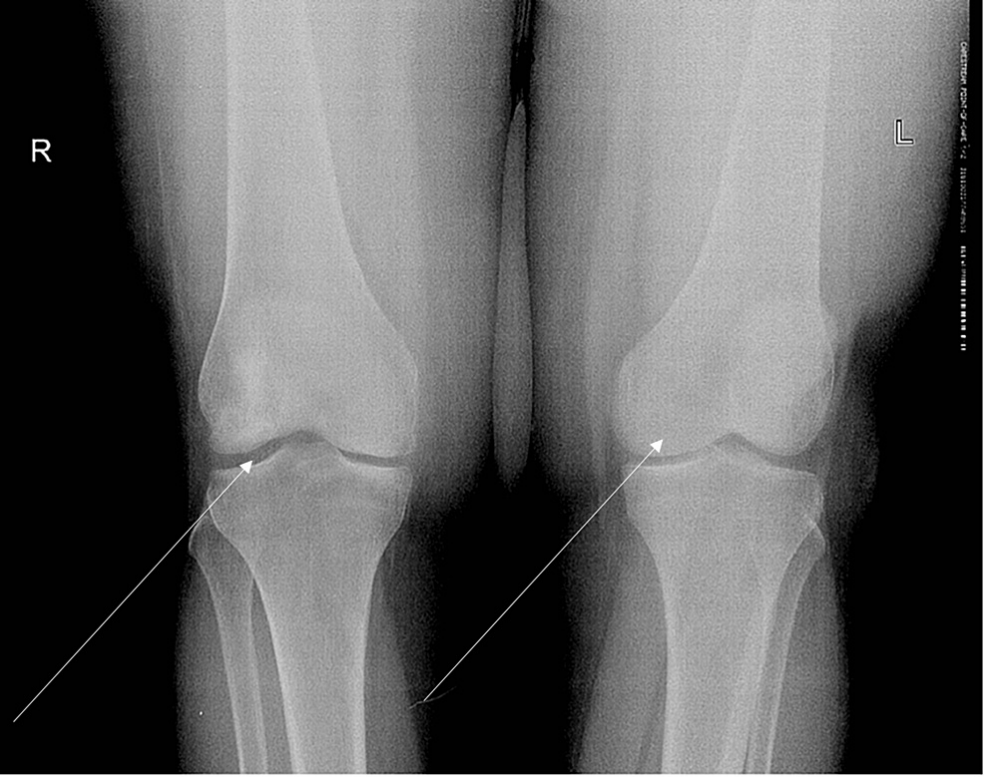
Anteroposterior radiograph of bilateral knees during the initial visit. Arrows indicate the area of possible avascular necrosis.

**Figure 2:**
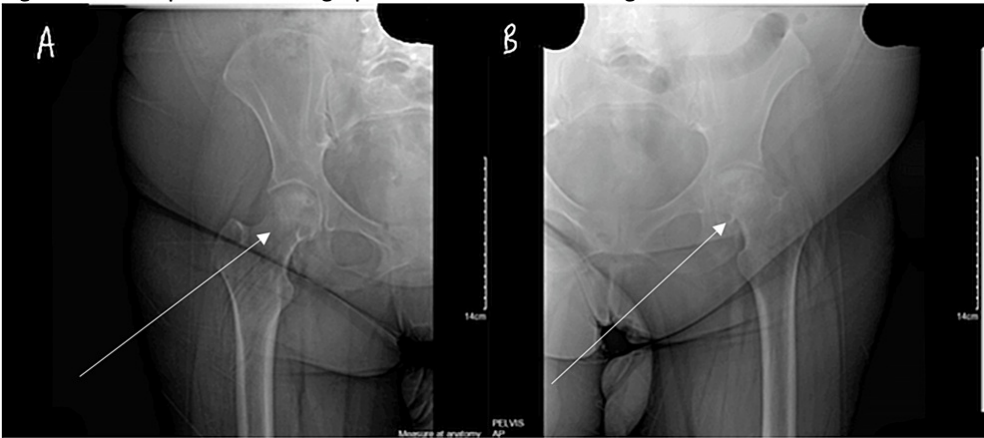
Anteroposterior radiographs of the left (A) and right hip (B). Arrows indicate the area of possible avascular necrosis.

**Figure 3:**
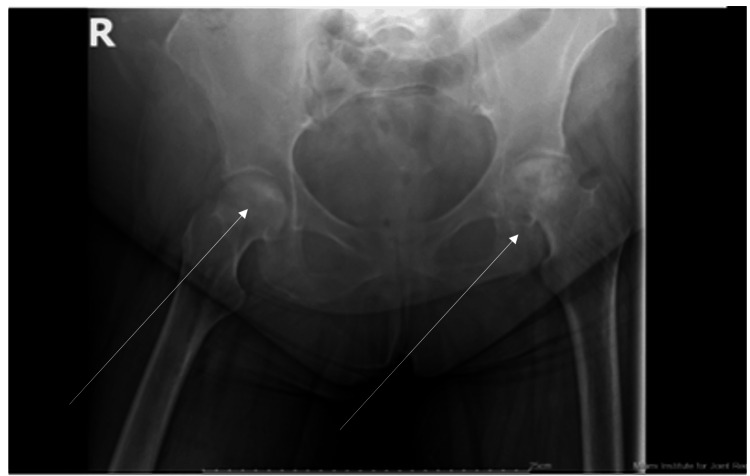
Anteroposterior radiograph of the pelvis. Arrows indicate the area of possible avascular necrosis.

**Figure 4:**
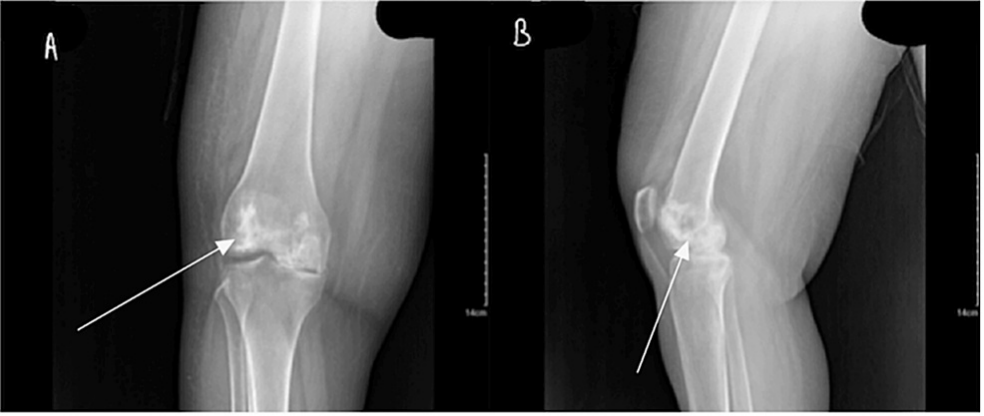
Anteroposterior (A) and lateral (B) radiographs of the right knee. Arrows indicate the area of avascular necrosis.

A 47-year-old female was seen at an outpatient orthopedic surgery clinic in May 2018 for left hip pain and bilateral knee pain that began gradually over years. The pain was rated 7/10 and worsened over the past two months. She developed the pain when she was stepping out of her room one day. The patient was a recipient of a kidney transplant for end-stage renal disease (ESRD) and had been on steroid medication for many years. She underwent a revision kidney transplant in 2016 and continued prednisone after the initial transplant and during her orthopedic follow-ups. She had no other pertinent medical, social, or family history. The physical examination of the right knee demonstrated a 0-110 range of motion with pain at the end of flexion and tenderness to palpation along the medial joint line. The physical examination of the left knee also demonstrated a 0-110 range of motion with pain at the end of flexion and tenderness to palpation along the medial joint line. There was pain in the left hip with internal rotation as well as reduced strength due to pain. The patient had brought in an MRI report of the left hip and bilateral knees done at an outside facility confirming multiple infarcts and AVN in the left hip, left femur, left knee, right femur, and right knee. Radiographs of the patient’s knees taken during the initial visit are shown in Figure 1. The patient also had osteoarthritis of bilateral knees. At the time of the visit, the patient was recommended partial weight bearing with a walker and physical therapy with a home exercise program. She was also given a range-of-motion brace for additional support. There was some improvement in the pain. The patient eventually returned in November 2020 to the outpatient clinic with excruciating right knee pain and a history of COVID-19 hospitalization. She most likely developed COVID-19 after May 2019 and before November 2020, although the exact hospital clinical course of her hospitalization was unobtainable. Nevertheless, she recalled being treated with steroid medication. Figures 2-4 represent the radiographs obtained during the outpatient follow-up in the clinic. The patient was a poor candidate for a total knee arthroplasty due to her prolonged corticosteroids, dialysis, ESRD, and possible further necessity of a kidney transplant. At this time, new MRIs of bilateral hips and knees demonstrated stage 4 osteonecrosis of the left hip while the right hip showed stage 2 osteonecrosis. There was also osteonecrosis along both femurs as well. We believed it was best for the patient to undergo a core decompression of the right hip to prevent exacerbation of osteoarthritis in that joint. Treatment with total joint replacement puts the patient at a higher risk of infection and complications due to her ESRD. Unfortunately, the patient was lost to follow-up and never returned to the clinic.

Case two

**Figure 5:**
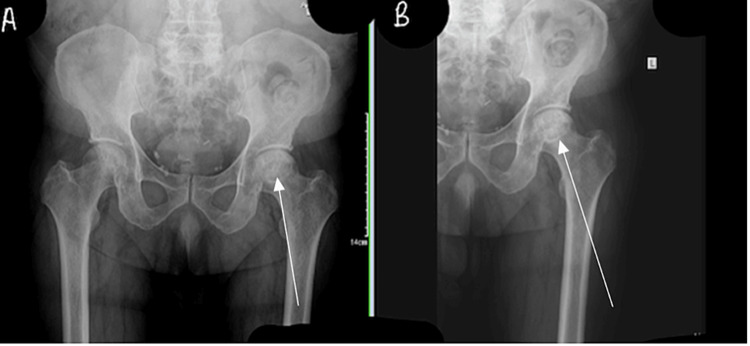
Anteroposterior radiograph of the pelvis (A) and left hip (B). Arrows show stage II avascular necrosis of the left hip without collapse of the left hip.

A 71-year-old man presented to an outpatient orthopedic surgery clinic for evaluation of left hip pain that was severe and constant. The pain was mostly in the groin and occurred during prolonged activity. His past medical history included hypertension. The patient was also previously hospitalized for COVID-19 pneumonia and treated with corticosteroids. However, he did not recall exactly when he was hospitalized or how much steroid medication he received. Hospital records were unobtainable. There was no surgical history or relevant family history. Physical examination demonstrated a full range of motion with pain on internal rotation. The patient was neurovascularly intact and had no leg length discrepancy. Radiographs showed a stage II AVN of the left hip without collapse (Figure 5). An MRI impression revealed that there was a large area of AVN in the left femoral head with marrow edema in the left femoral head and proximal femur. There was no flattening of the left femoral head or secondary osteoarthritic changes. We believed at that time the best course of action was to proceed with core decompression. Surgery was performed in the following weeks, and the patient’s pain improved. He followed up in the clinic and was prescribed anti-inflammatory medication and physical therapy. The patient’s overall pain in the left hip improved.

Case three

**Figure 6:**
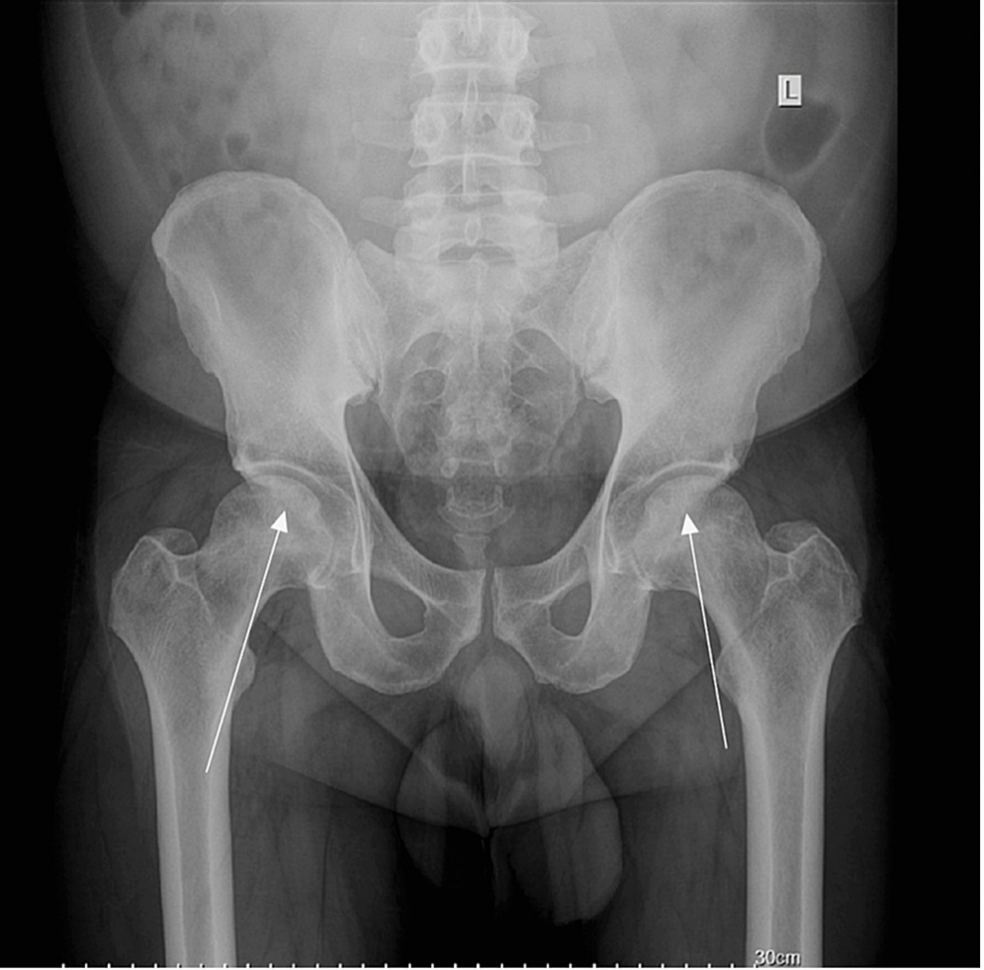
Anteroposterior radiograph of the pelvis. Arrows indicate stage II bilateral femoral head and left distal femur avascular necrosis without collapse.

**Figure 7:**
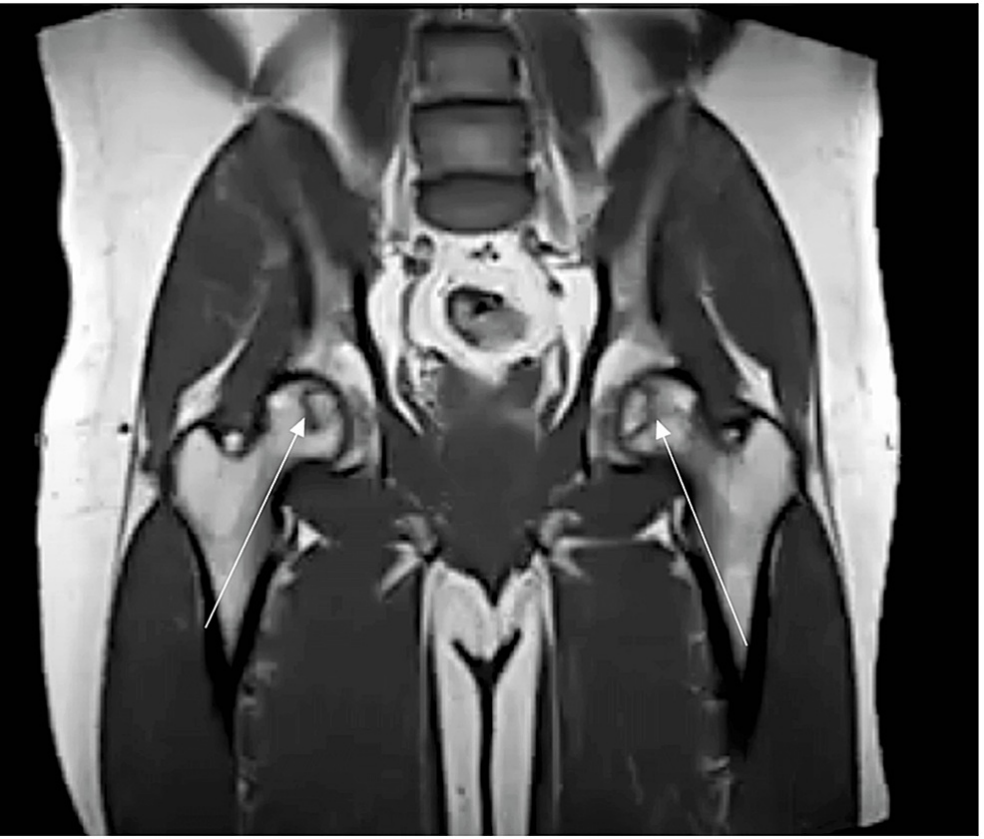
MRI of the anteroposterior pelvis. Arrows indicate stage II bilateral femoral head and left distal femur avascular necrosis without collapse on MRI.

A 49-year-old male, with a past medical history of lumbar radiculopathy presented to an outpatient clinic with left hip pain that began gradually at work and worsened over the past month with physical activity. The pain had been present for a while. He ambulated using a walker and had a past medical history of high-dose steroid treatment during hospital admission for COVID-19. He was hospitalized for COVID-19 in 2020, two years before his initial outpatient visit. He could not recall the exact date or treatment timeline. The specific dosage was unknown and hospital records were unobtainable. Prior to this initial visit, the patient was worked up for AVN of the left hip and left distal femur by another physician. The patient had continued pain in the left hip and groin area. The patient also had unrelated back pain with tingling down his lower extremities. He denied bowel or bladder problems. Physical examination demonstrated pain on palpation of the groin area. There was also pain reproduced with internal and external hip rotation. The patient was neurovascularly intact with 5/5 muscle strength in all planes. Diagnostic imaging confirmed the presence of stage II bilateral femoral head and left distal femur AVN without collapse (Figures 6, 7). Therefore, it was in the best interest of the patient that we proceeded with bilateral core decompression procedures to reduce the pressure inside the femoral head and prevent collapse. The patient was then scheduled for surgery the following week and followed up in the outpatient clinic. Anti-inflammatory medication and physical therapy were prescribed. The patient eventually returned with no pain.

## Discussion

Two patients with left hip AVN presented to an outpatient clinic after steroid treatment for COVID-19 pneumonia. Both had stage II AVN of the left hip and were treated with core decompression. This procedure alleviates the increased pressure in the femoral head and prevents collapse and worsening of osteoarthritis in the future. Both patients had improved pain. The third patient had multiple infarcts of AVN in the left hip and bilateral knees which eventually progressed to involve the bilateral femurs, hips, and knees. The patient had excruciating pain mainly in the right knee and left hip; however, treatment with total joint replacement was contraindicated due to her history of ESRD. Therefore, core decompression of the right hip was recommended to prevent further worsening of AVN and femoral head collapse. The goal of this paper is to speculate on a possible link between COVID-19 and AVN, including steroid treatment and/or COVID-19 virulence.

Although these instances of AVN could be attributed to different causes, it raises the question about the relationship between steroid treatment and COVID-19 in the development of AVN. As previously stated, COVID-19 causes a hypercoagulable state which increases the risk of thrombosis and initiation of the AVN cascade. Therefore, it can be hypothesized that COVID-19 potentially exacerbates corticosteroid-induced AVN. The presentation of corticosteroid-induced AVN in COVID-19 patients may occur earlier than the recommended time frame and with a lower dose [7,11]. According to a case series by Agarwala et al., COVID-19 patients who suffer from AVN were exposed to lower cumulative doses of corticosteroid medication necessary to cause AVN than patients taking steroid medication without COVID-19 infection [11]. No exact cumulative dosage associated with the increased risk of AVN exists; however, some authors suggest that a cumulative dose of 2,000 mg can cause AVN [12]. Moreover, studies show that cumulative dosage of steroid medication may be a better predictor for AVN than daily dosage [13]. The patients in that study had a mean cumulative corticosteroid dosage of 758 mg and developed AVN at 58 days, which is earlier than most cases of AVN after steroid administration (6-12 months) [7,11]. This suggests that the combination of corticosteroid use and COVID-19 may hasten the onset of AVN compared to corticosteroid use alone in patients without COVID-19. Moreover, COVID-19 is known to produce coagulopathies in patients, specifically hypercoagulability, that may contribute to venous thrombosis and AVN [10]. Therefore, patients treated with corticosteroids for COVID-19 may be more likely to develop AVN than patients treated with corticosteroids for different conditions.

One study by Dhanesekararaja et al. analyzed data from 22 patients treated with corticosteroids for COVID-19 and developed osteonecrosis of the femoral head [14]. According to their results, the mean time to diagnosis was 39.3 days, the average onset of symptoms after COVID-19 infection was 7.5 months, the average cumulative dose of methylprednisolone was 811 mg, and the average duration of steroid intake was 2.8 weeks [15]. They concluded that while the most common presentation of AVN is similar to classical osteonecrosis of the femoral head, some patients can have an acute and aggressive presentation with rapid destruction. Furthermore, their patients had a low cumulative dose of steroids making COVID-19 vasculitis a possible factor in the development of osteonecrosis of the femoral head [13]. Other studies have also demonstrated osteonecrosis of the jaw, maxilla, and knee as a result of COVID-19 and corticosteroid treatment [3,5,16].

The three cases mentioned here argue for a possible association between COVID-19, corticosteroid treatment, and AVN. Age, however, may be a confounding variable as increased age increases the risk of AVN [16]. Furthermore, specific data regarding treatment type, dosage, and duration were unobtainable and steroid treatment history was obtained anecdotally. Nevertheless, based on the history and presentation of the patient previously described, it is believed that a relationship between COVID-19 and AVN may be present. Further studies should include retrospective reviews or case-control studies identifying the specific differences in steroid treatment dosage and duration of patients with and without COVID-19-associated AVN.

## Conclusions

Although no studies directly compare the effect of corticosteroid use in patients with COVID-19 versus those without COVID-19, based on the current literature, it can be hypothesized that the combination of COVID-19 and corticosteroid treatment may be associated with an increased risk of AVN in patients despite comorbidities. Our case series supports this hypothesis. Therefore, providers should proceed with caution when using corticosteroids to treat COVID-19 patients and manage the dosage appropriately. Risk stratification based on the severity of COVID-19 infection and corticosteroid intake can help identify patients who are at a higher risk of osteonecrosis and can be managed accordingly. Regular evaluation of hip pain during follow-up and increased awareness may help identify AVN early and reduce the risk of degenerative joint disease. Future studies should focus on performing retrospective cohort reviews or case-control studies that directly compare AVN parameters in patients with COVID-19 and corticosteroid treatment to corticosteroid treatment alone.

## References

[1] Shah KN, Racine J, Jones LC, Aaron RK (2015). Pathophysiology and risk factors for osteonecrosis. Curr Rev Musculoskelet Med.

[2] Jones LC, Mont MA (2022). Clinical manifestations and diagnosis of osteonecrosis (avascular necrosis of bone). UpToDate.

[3] Angulo-Ardoy M, Ureña-Aguilera Á (2021). Knee osteonecrosis after COVID-19. Fam Pract.

[4] Ghosh S, Gupta SS, Mehta N, Khodaiji S (2021). COVID-19-associated bone marrow necrosis-a case report. Indian J Radiol Imaging.

[5] Hasan A, Alraisi S (2021). MRONJ and COVID-19 caution. Br Dent J.

[6] Zhang S, Wang C, Shi L, Xue Q (2021). Beware of steroid-induced avascular necrosis of the femoral head in the treatment of COVID-19-experience and lessons from the SARS epidemic. Drug Des Devel Ther.

[7] Banerjee I, Robinson J, Sathian B (2021). Corticosteroid induced avascular necrosis and COVID-19: the drug dilemma. Nepal J Epidemiol.

[8] Disser NP, De Micheli AJ, Schonk MM (2020). Musculoskeletal consequences of COVID-19. J Bone Joint Surg Am.

[9] Akbarialiabad H, Taghrir MH, Abdollahi A (2021). Long COVID, a comprehensive systematic scoping review. Infection.

[10] Gómez-Mesa JE, Galindo-Coral S, Montes MC, Muñoz Martin AJ (2021). Thrombosis and coagulopathy in COVID-19. Curr Probl Cardiol.

[11] Agarwala SR, Vijayvargiya M, Pandey P (2021). Avascular necrosis as a part of 'long COVID-19'. BMJ Case Rep.

[12] Jones JP (2001). Osteonecrosis. Arthritis and Allied Conditions: A Textbook of Rheumatology.

[13] Chan KL, Mok CC (2012). Glucocorticoid-induced avascular bone necrosis: diagnosis and management. Open Orthop J.

[14] Dhanasekararaja P, Soundarrajan D, Kumar KS, Pushpa BT, Rajkumar N, Rajasekaran S (2022). Aggressive presentation and rapid progression of osteonecrosis of the femoral head after COVID-19. Indian J Orthop.

[15] Mañón VA, Balandran S, Young S, Wong M, Melville JC (2022). COVID-associated avascular necrosis of the maxilla-a rare, new side effect of COVID-19. J Oral Maxillofac Surg.

[16] Wang WT, Li YQ, Guo YM (2019). Risk factors for the development of avascular necrosis after femoral neck fractures in children: a review of 239 cases. Bone Joint J.

